# Ultrafast sub–30-fs all-optical switching based on gallium phosphide

**DOI:** 10.1126/sciadv.aaw3262

**Published:** 2019-06-14

**Authors:** Gustavo Grinblat, Michael P. Nielsen, Paul Dichtl, Yi Li, Rupert F. Oulton, Stefan A. Maier

**Affiliations:** 1 The Blackett Laboratory, Department of Physics, Imperial College London, London SW7 2AZ, UK.; 2 Departamento de Física, FCEN, IFIBA-CONICET, Universidad de Buenos Aires, C1428EGA Buenos Aires, Argentina.; 3 School of Photovoltaic and Renewable Energy Engineering, University of New South Wales, Sydney, NSW 2052, Australia.; 4 Chair in Hybrid Nanosystems, Nanoinstitut München, Fakultät für Physik, Ludwig-Maximilians-Universität München, 80539 München, Germany.

## Abstract

Gallium phosphide (GaP) is one of the few available materials with strong optical nonlinearity and negligible losses in the visible (λ > 450 nm) and near-infrared regime. In this work, we demonstrate that a GaP film can generate sub–30-fs (full width at half maximum) transmission modulation of up to ~70% in the 600- to 1000-nm wavelength range. Nonlinear simulations using parameters measured by the *Z*-scan approach indicate that the transmission modulation arises from the optical Kerr effect and two-photon absorption. Because of the absence of linear absorption, no slower free-carrier contribution is detected. These findings place GaP as a promising ultrafast material for all-optical switching at modulation speeds of up to 20 THz.

## Teaser

Pump-probe spectroscopy of a gallium phosphide film shows potential for efficient ultrafast sub–30-fs all-optical switching.

## INTRODUCTION

Integrated photonics has experienced huge growth in recent decades, with constantly expanding applications in telecommunications and computing ranging from long- and short-range data transfer (*1*) to the generation (*2*), modulation (*3*), and detection (*4*) of data signals. However, challenges remain as the core promise of integrated photonics, high-speed all-optical switching, still needs to be realized to replace electronics in information processing: first is to increase the modulation speed of integrated photonics (*3*) and then to extend the operating wavelengths into the visible (*5*) and mid-infrared (*6*) regime. In addition, as terahertz switching times require the use of ultrashort pulses, there is a need to understand how the broadband nature of ultrashort pulses affects all-optical signal modulation approaches.

Gallium phosphide (GaP) is an important photonic material, having long been used as the active material in green light-emitting diodes (*7*). More recent nanoscale demonstrations of GaP devices in light-producing applications have included harmonic generation (*8*, *9*) and nanoparticle-based fluorescence enhancement (*9*, *10*). GaP is an indirect bandgap (2.26 eV) semiconductor with a high refractive index (*n* > 3) across the visible and near-infrared regime (*11*), along with an associated high third-order nonlinearity (*12*), and is transparent between 450 nm and 11 μm. Moreover, as a noncentrosymmetric crystal, GaP has a large second-order nonlinearity and a nonzero piezoelectric coefficient, making the material well suited to future integrated photonics applications across the visible, near-infrared, telecommunications, and mid-infrared wavelengths. Wafer-scale fabrication of GaP-on-insulator has recently been demonstrated along with a variety of passive photonic devices (*13*). In this article, we present ultrafast all-optical switching from a GaP film in the 600- to 1000-nm wavelength range with characteristic switching times below 30 fs and transmission modulations as high as ~70%. This suggests that GaP could be a promising material for all-optical computing and ultrafast modulators.

## RESULTS AND DISCUSSION

**Fig. 1:**
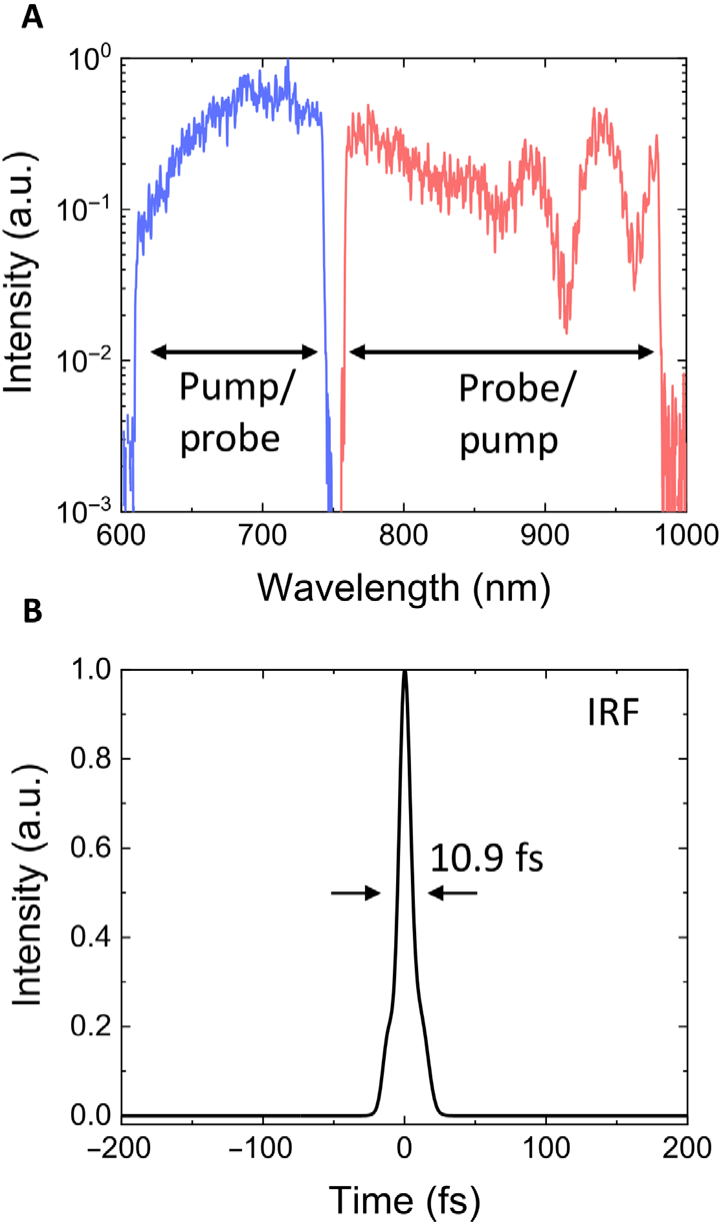
Ultrafast pump and probe pulses. (**A**) Spectra of the pulsed laser beams used for the nondegenerate pump-probe experiments, used interchangeably as pump and probe beams. (**B**) IRF of the pump-probe technique, computed as the temporal convolution between the two different trains of pulses. a.u., arbitrary units.

A commercial double-side polished 350-μm-thick GaP (100) wafer was studied through nondegenerate ultrafast pump-probe spectroscopy. The spectra of the two laser beams used for the experiment are shown in Fig. 1A, composed of short (610 to 745 nm) and long (760 to 980 nm) wavelength regions, respectively, and are interchangeably used as pump and probe beams. The linear polarization of the beams was chosen to be within the GaP (100) plane. The corresponding instrument response function (IRF) of the technique is shown in Fig. 1B, defined as the convolution between the intensity envelopes of the measured temporal responses of the two pulsed beams, evaluated at the position of the sample through interferometric–FROG (frequency-resolved optical gating). The pump and probe pulses were measured to have a pulse width of ~7 fs, with a corresponding IRF of 10.9 fs FWHM (full width at half maximum). (More details about the pump-probe experimental setup and pulse characterization can be found in Materials and Methods.)

**Fig. 2:**
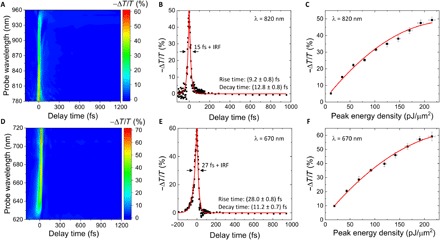
Ultrafast pump-probe results. (**A**) Differential transmittivity spectra of the GaP sample as a function of pump-probe delay time, when pumped with the short-wavelength beam. (**B**) Cross-section of the data plotted in (A) at λ = 820 nm. Solid red lines in the graph correspond to a fit considering the convolution between the IRF and exponential functions. (**C**) Pump peak energy density dependence of −Δ*T*/*T* at λ = 820 nm when pumping with the short-wavelength beam. (**D** to **F**) As (A) to (C), respectively, but pumping with the long-wavelength beam and with the cross-section and power dependence taken at λ = 670 nm.

Differential transmittivity (−Δ*T*/*T*) results are shown in Fig. 2A, as measured by pumping the GaP sample with the short-wavelength beam at a peak energy density of 220 pJ/μm^2^, using the long-wavelength beam as the probe, with a 30:1 pump/probe power ratio, as a function of probe wavelength and pump-probe delay time (*t*). By analyzing the optical signal after interaction with the sample, the time dynamics of each composite wavelength of the probe could be studied without altering the pulse duration at the position of the sample. A strong reduction in the transmissivity can be observed around *t* = 0 fs, reaching values close to 60% modulation. The magnitude of the response is found to decrease toward the long-wavelength edge, which corresponds to the furthest wavelengths from the pump pulse. Because GaP presents no linear absorption in this spectral region, free-carrier effects are expected to be negligible, explaining why no slower response due to free-carrier recombination is observed in the picosecond range. This nearly instantaneous change in transmissivity, which drops to 0% within 100 fs, is thought to arise from a modification in the GaP refractive index by its third-order nonlinear response via the optical Kerr effect and two-photon absorption, as demonstrated later in the article. Note that the presence of fringes near the short-wavelength edge in Fig. 2A is a consequence of pump spectral broadening due to four-wave mixing, which causes interference with the probe signal. The response of the semiconductor at λ = 820 nm is shown in Fig. 2B. To extract rise and decay times, we fitted the data with exponential growth and decay functions for *t* < 0 fs and *t* > 0 fs, respectively, convolved with the IRF, which was approximated by a Gaussian profile. A rise time of (9.2 ± 0.8) fs and a decay time of (12.8 ± 0.8) fs were attained, with an associated FWHM of ~15 fs and a full width at tenth maximum (FWTM) of ~50 fs. Such an ultrafast response would allow all-optical modulation bandwidths as large as 20 THz. We note that this modulation bandwidth is more than one order of magnitude broader than that expected from ITO (indium tin oxide) in its epsilon-near-zero region (*14*–*16*). A film of ITO has been demonstrated to produce a comparable modulation in transmissivity of ~68% at the same peak pump energy density but with a temporal width of 650 fs at 10% of maximum response (*16*). Moreover, unlike using the lossy and narrowband epsilon-near-zero region of ITO, using GaP both reduces a potential device’s insertion loss and guarantees broadband operation. Other examples of efficient optical switching devices using the optical Kerr effect and two-photon absorption usually rely on high Q factor resonances, exhibiting a temporal response that is limited by the cavity lifetime to the picosecond (*17*) or even nanosecond (*18*) range. The dependence of −Δ*T*/*T* as a function of the pump power is depicted in Fig. 2C, revealing that the transmission modulation saturates. Given that the nonlinear effects in the experiment should occur entirely within the Rayleigh range (~20 μm) of the Gaussian focus, similar results should be possible for GaP films considerably thinner than the one studied here (350 μm thickness). The effective volume of interaction between the sample and the laser beams is estimated to be as small as <35 μm^3^.

The results measured, when pumping the sample with the long-wavelength beam at a peak energy density of 220 pJ/μm^2^ and probing with the short-wavelength beam, with a 30:1 pump/probe power ratio, can be seen in Fig. 2 (D to F). A similar behavior to that observed in Fig. 2 (A to C) is found. In Fig. 2D, a maximum transmissivity modulation of ~70% is obtained, showing a similar interference effect to that in Fig. 2A at the wavelengths closest to the pump spectral region and presenting a reduced response at the wavelengths furthest from those composing the pump. The temporal trace result at λ = 670 nm is shown in Fig. 2E. In this case, rise and decay times of (28.0 ± 0.8) fs and (11.2 ± 0.7) fs were attained, respectively, with associated FWHM of ~27 fs and FWTM of ~90 fs. Regarding the power dependence of −Δ*T*/*T*, shown in Fig. 2F, a comparable saturation effect to that in Fig. 2C can be observed. It should be mentioned that the material response time seen for both pump-probe configurations is not nearly transform limited [third-order nonlinear effects should be limited to a Heisenberg uncertainty lifetime of only a few femtoseconds or less (*19*)]. We attribute this extended temporal response to the growing group velocity dispersion inside the material at photon energies near that of GaP’s bandgap, as has been observed with highly nondegenerate two-photon absorption (*20*). The ultrafast pulses stretch in time near the bandgap wavelength (~550 nm), producing a wider temporal response at the shortest wavelengths, as seen in Fig. 2D. While low wavelengths still participate in the differential transmissivity signal probed at high wavelengths, they contribute with a small relative weight to the pump spectrum (see the blue curve in Fig. 1A), and therefore, an overall faster temporal response is measured in Fig. 2A.

To support the experimental results, we conducted nonlinear pump-probe simulations including the optical Kerr effect and two-photon absorption using the commercial software Lumerical FDTD Solutions (see Materials and Methods and the Supplementary Materials for simulation specifics). The optical Kerr effect is a third-order nonlinear effect that results from the response of bound electrons to the incoming light, inducing a nonlinear polarization in the medium that modifies the real component of the refractive index of the material. Because no direct transfer of carriers into excited states occurs, there is no time limitation for the excitation or relaxation processes, and therefore, the phenomenon can take place in a virtually instantaneous manner. Two-photon absorption requires the simultaneous absorption of one-pump and one-probe photons (nondegenerate case) and modifies the imaginary component of the refractive index. In the experiment, once the pump and probe pulses are no longer temporally overlapped, both nonlinear phenomena cease to exist, producing a nearly instantaneous decay in the probe signal. We note, however, that degenerate two-photon absorption of the pump also occurs and, together with the nondegenerate process, produces a small number of free carriers, in contrast to the optical Kerr effect. Nevertheless, their (slow) recombination mechanisms do not cause an appreciable differential transmissivity response in the measurement (i.e., there is no long-lasting tail in the pump-probe decay signal in Fig. 2). This is because neither the probe photon energy nor power is large enough to produce a sufficiently high electron-hole pair density (either through linear or degenerate nonlinear absorption) to sensibly identify persisting changes in the electronic distribution.

In a Kerr medium, the complex refractive index of the material can be approximated as $\tilde{n}=(n_{0}+ik_{0})+I(n_{2}+ik_{2})$, where *n*_0_ and *k*_0_ are the low-intensity real and imaginary parts of the refractive index, respectively; *I* is the intensity of the pump beam; and *n*_2_ and *k*_2_ are the nonlinear index and nonlinear absorption, respectively. The parameter *k*_2_ relates to the two-photon absorption coefficient, β_TPA_, through the equation *k*_2_ = (λβ_TPA_)/(4π). To estimate *n*_2_ and β_TPA_, we performed degenerate *Z*-scan measurements in the 600- to 1000-nm range (see Materials and Methods and the Supplementary Materials for experimental details of the *Z*-scan technique and corresponding results). Average values of *n*_2_ of 9.4 × 10^−18^ m^2^/W and β_TPA_ of 6.4 × 10^−11^ m/W were attained across the wavelength regime. However, given that the values of nondegenerate *n*_2_ and β_TPA_ relevant to the pump-probe experiments cannot be determined from the degenerate ones (*21*), we analyzed possible values in the vicinity of the measured range that would lead to the measured differential transmissivity signals.

**Fig. 3:**
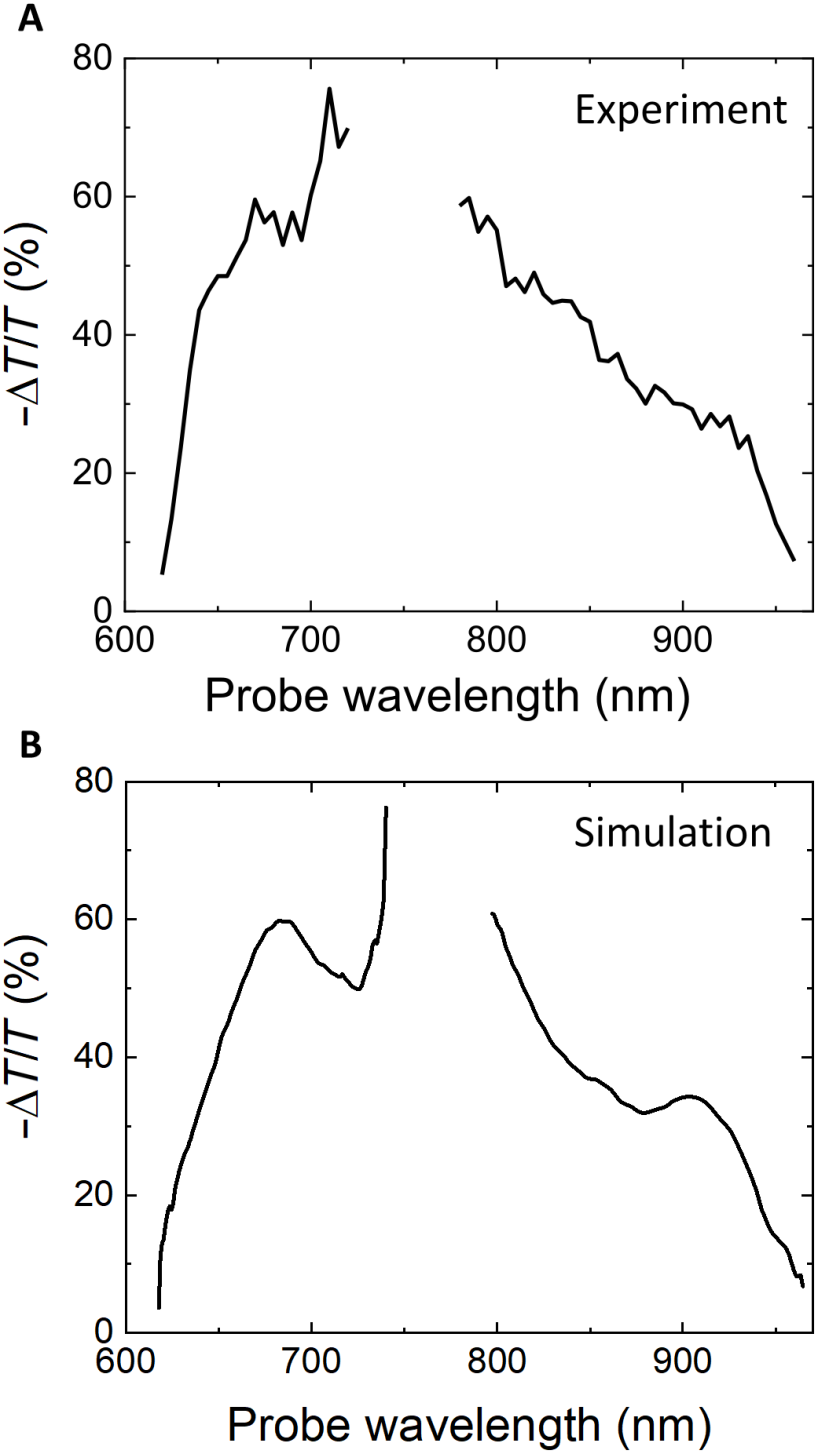
Comparison of experimental results and nonlinear numerical simulations. Experimental (**A**) and simulated (**B**) differential transmittivity spectra of the GaP sample at *t* = 0 fs, considering both short- and long-wavelength pumping configurations.

The obtained experimental and simulated transmissivity changes as a function of probe wavelength at *t* = 0 fs for both pumping conditions at a pump peak energy density of 220 pJ/μm^2^ are shown, respectively, in Fig. 3 (A and B). Very good agreement is found when considering *n*_2_ = 2 × 10^−18^ m^2^/W and β_TPA_ = 3 × 10^−11^ m/W for short-wavelength pumping and *n*_2_ = 4 × 10^−18^ m^2^/W and β_TPA_ = 8 × 10^−11^ m/W for long-wavelength pumping. We note that these values, which are comparable to those measured by the *Z*-scan method, should be regarded as effective ones, because the nondegenerate nonlinear parameters *n*_2_ and β_TPA_ vary depending on the pair of pump and probe wavelengths. In both cases, the peak effective change in the complex refractive index is $\Delta \tilde{n}<0.1+i0.1$. Such a relatively high change in the complex refractive index is supported by the evidence of having entered a saturation regime as seen in Fig. 2 and is well within what has been demonstrated in other Kerr materials such as ITO (*14*–*16*). Regarding the role of these coefficients in the observed transmission reduction, *n*_2_ is found to contribute via an increased reflection at the incident air/GaP interface. β_TPA_ is deduced to be the main responsible factor, causing cumulative nonlinear absorption inside the semiconductor film throughout the Rayleigh range, with an estimated contribution to the measured response of about 80%.

In summary, using a pump-probe technique with a ~10-fs resolution, we studied the ultrafast nonlinear dynamics of crystalline GaP in the 600- to 1000-nm wavelength range. Differential transmissivity measurements showed that this semiconductor can produce sub–30-fs transmissivity modulations of up to ~70% in magnitude in a <35-μm^3^ volume, as a result of nearly instantaneous changes in the complex refractive index due to optical Kerr effect and two-photon absorption. These findings suggest that suitable nanostructuring of this material to confine the incident electric field inside the semiconductor would enable the possibility of efficient ultrafast all-optical signal processing on the nanometer scale.

## MATERIALS AND METHODS

### Pump-probe experiments

A pulsed Yb:KGW PHAROS laser system coupled to an ORPHEUS collinear optical parametric amplifier with a LYRA wavelength extension (pulse duration, ~180 fs; repetition rate, 100 kHz; wavelength range, 315 to 2600 nm; Light Conversion Ltd.) was used to generate supercontinuum light by focusing 1120-nm wavelength at 200-mW average power onto a 5-mm-thick sapphire plate using a plano-convex lens of 5-cm focal length. The supercontinuum beam was collimated with a spherical mirror and split into 610- to 745-nm and 760- to 980-nm spectral components using dichroic beam splitters. The wide spectrum beams were coupled to a MIIPS (multiphoton intrapulse interference phase scan) device (MIIPSBox640-P, Biophotonic Solutions Inc.), able to compress the pulses in time down to bandwidth-limited ~7-fs pulses at the position of the sample, as verified through interferometric-FROG autocorrelation measurements performed with the same device. More details on the ultrashort pulse creation can be found published elsewhere (*22*). A motorized optical delay line by Thorlabs was used to introduce controlled time differences between the different trains of pulses with <1-fs accuracy. The two pulsed beams, used interchangeably as pump and probe beams, were focused into the sample using a metal objective of 0.5 numerical aperture. A spectrograph (PI Acton SP2300, Princeton Instruments) coupled to a low-noise Si photodiode (picowatt photoreceiver series PWPR-2K, FEMTO) was used for spectral characterization of the probe light transmitted by the sample. The measurements were carried out with lock-in detection by modulating the pump beam at <1-kHz frequency using an optical chopper.

### *Z*-scan measurements

The third-order nonlinearities of the GaP sample in the 600- to 1000-nm wavelength range were estimated with the single-beam *Z*-scan technique, using the same setup as described elsewhere (*23*). The laser beam from the tunable laser system mentioned above was focused onto the sample with a 150-mm plano-convex lens (Rayleigh range of 2.2 ± 0.3 mm), and the transmitted light was recorded with a Thorlabs DCC1545M CMOS (complementary metal-oxide semiconductor) camera. In the long-wavelength end (900 to 1000 nm), the nonlinear absorption coefficient was determined using a 125-mm lens to improve the signal-to-noise ratio. The sample was moved through the lens focus using a Thorlabs PT1/M-Z8 motorized stage. For each position of the sample, the transmitted intensity distribution was registered, and the open and closed *Z*-scan traces were obtained by summing over all camera pixels and over a reduced circular region in the center containing 2% of the total intensity, respectively. The normalized *Z*-scan data were fitted using standard formulae from the literature (*24*, *25*). *Z*-scan results can be found in the Supplementary Materials.

### Numerical simulations

The simulations were performed with the finite-difference time-domain (FDTD) technique using the commercial software Lumerical FDTD Solutions (more details on the simulations can be found in the Supplementary Materials). The linearly polarized pulses were inputted via Gaussian focus using a numerical aperture of 0.5 and with the focal position 3 μm below the air/GaP interface, in agreement with the experimental conditions. Perfectly matched layers were used as the simulation boundary conditions to avoid reflections. The experimental spectra for the pump/probe pulses (as seen in Fig. 1A) were imported into the FDTD simulations as custom sources, which resulted in ripples in the simulated differential transmissivity curves. These were removed by smoothing through adjacent averaging. Note that replacing the custom sources by standard Gaussian spectra sources with the same temporal duration resulted in smooth differential transmissivity curves but gave poor agreement with the experimental curves.

Ellipsometry measurements were used to determine the linear optical properties of GaP (refer to the Supplementary Materials for ellipsometry data), while the nonlinear properties, an instantaneous Kerr effect and two-photon absorption, were implemented into the FDTD simulations following the formulism of Suzuki (*26*). By scanning the Kerr index and two-photon absorption coefficients for GaP across the wavelength spectrum as determined by the *Z*-scan method, approximate matches for the experimental differential transmissivity curves were found, as seen in Fig. 3.

## Supplementary Material

http://advances.sciencemag.org/cgi/content/full/5/6/eaaw3262/DC1

Download PDF

## SUPPLEMENTARY MATERIALS

Supplementary material for this article is available at http://advances.sciencemag.org/cgi/content/full/5/6/eaaw3262/DC1 Ellipsometry measurements *Z*-scan measurements Numerical simulations Fig. S1. Real and imaginary parts of GaP refractive index as a function of wavelength as measured by ellipsometry. Fig. S2. *Z*-scan results of a double-side polished 350-μm-thick GaP sample. Fig. S3. Schematic representation of the numerical simulation volume depicting the ultrashort pulse injection plane, the nominal focus position, and the simulation length. Fig. S4. Linear numerical simulations. Fig. S5. Simulation of pump and probe pulses. Reference (*27*)
