# Supplementary material for: Ultrafast sub–30-fs all-optical switching based on gallium phosphide
Source: Sci Adv. 2019 Jun 14;5(6):eaaw3262. doi: 10.1126/sciadv.aaw3262 (PMC6570513; doi:10.1126/sciadv.aaw3262)
Supplement: http://advances.sciencemag.org/cgi/content/full/5/6/eaaw3262/DC1 [file supp_5_6_eaaw3262__index.html]

Science Advances | Science Advances

## Supplementary Materials

**This PDF file includes:**

- Ellipsometry measurements
- *Z*-scan measurements
- Numerical simulations
- Fig. S1. Real and imaginary parts of GaP refractive index as a function of wavelength as measured by ellipsometry.
- Fig. S2. *Z*-scan results of a double-side polished 350-μm-thick GaP sample.
- Fig. S3. Schematic representation of the numerical simulation volume depicting the ultrashort pulse injection plane, the nominal focus position, and the simulation length.
- Fig. S4. Linear numerical simulations.
- Fig. S5. Simulation of pump and probe pulses.
- Reference (*27*)

Download PDF

**Files in this Data Supplement:**

- Adobe PDF - aaw3262\_SM.pdf
