# Supplementary material for: Ultrafast sub–30-fs all-optical switching based on gallium phosphide
Source: Sci Adv. 2019 Jun 14;5(6):eaaw3262. doi: 10.1126/sciadv.aaw3262 (PMC6570513; doi:10.1126/sciadv.aaw3262)
Supplement: Download PDF [file aaw3262_SM.pdf]

Science Advances
AAAS

advances.sciencemag.org/cgi/content/full/5/6/eaaw3262/DC1

Supplementary Materials for

Ultrafast sub–30-fs all-optical switching based on gallium phosphide

Gustavo Grinblat*, Michael P. Nielsen, Paul Dichtl, Yi Li, Rupert F. Oulton, Stefan A. Maier

*Corresponding author. Email: g.grinblat@imperial.ac.uk

Published 14 June 2019, *Sci. Adv.* **5**, eaaw3262 (2019)
DOI: 10.1126/sciadv.aaw3262

This PDF file includes:

Ellipsometry measurements
Z-scan measurements
Numerical simulations
Fig. S1. Real and imaginary parts of GaP refractive index as a function of wavelength as measured by ellipsometry.
Fig. S2. *Z*-scan results of a double-side polished 350-μm-thick GaP sample.
Fig. S3. Schematic representation of the numerical simulation volume depicting the ultrashort pulse injection plane, the nominal focus position, and the simulation length.
Fig. S4. Linear numerical simulations.
Fig. S5. Simulation of pump and probe pulses.
Reference (*27*)

Ellipsometry measurements

Ellipsometry measurements of the GaP sample were carried out with a VASE ellipsometer. The obtained real (*n*) and imaginary (*k*) components of GaP refractive index in the 250-1200 nm wavelength range are shown in Fig. S1, which were used for the numerical simulations.

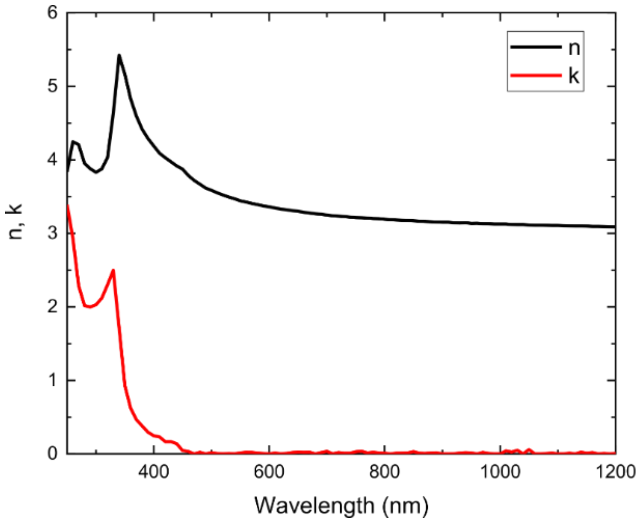

Fig. S1. Real and imaginary parts of GaP refractive index as a function of wavelength as measured by ellipsometry.

Z-scan measurements

The normalized Z-scan data, measured using the setup described elsewhere (*23*), were fitted using first-order expressions of the standard formulae from the literature (*25*)

$$T_{open}(z) = 1 - \frac{\beta_{TPA} I_0 L_{eff}}{2\sqrt{2}(z^2+1)} \quad \text{Eq. S1}$$

and (*24*)

$$T_{closed}(z) = 1 + \frac{4z\Delta\phi_0 - (z^2+3)\beta_{TPA} I_0 L_{eff}}{\sqrt{2}(z^2+1)(z^2+9)} \quad \text{Eq. S2}$$

where z is the position of the sample relative to the focus, I_0 is the peak intensity of the laser, L_{eff} is the effective length of the sample, β_{TPA} is the two-photon absorption coefficient, and

$\Delta\phi_0 = k\gamma I_0 L_{eff}$ is the peak on-axis nonlinear phase shift.

Representative open and closed aperture degenerate Z-scan measurements of the GaP sample carried out at λ = 850 nm and I_0 = 14 TW/m^2, can be seen in Fig. S2A with corresponding fits. Figs. S2B and S2C show the results obtained for n_2 and β_{TPA} in the whole 600-1000 nm wavelength range. Based on these findings, values in the vicinity of the range 5-16×10^{-18} m^2/W and 0.4-20×cm/GW were considered for the effective nonlinear parameters n_2 and β_{TPA}, respectively, relevant to the nondegenerate pump-probe experiments.

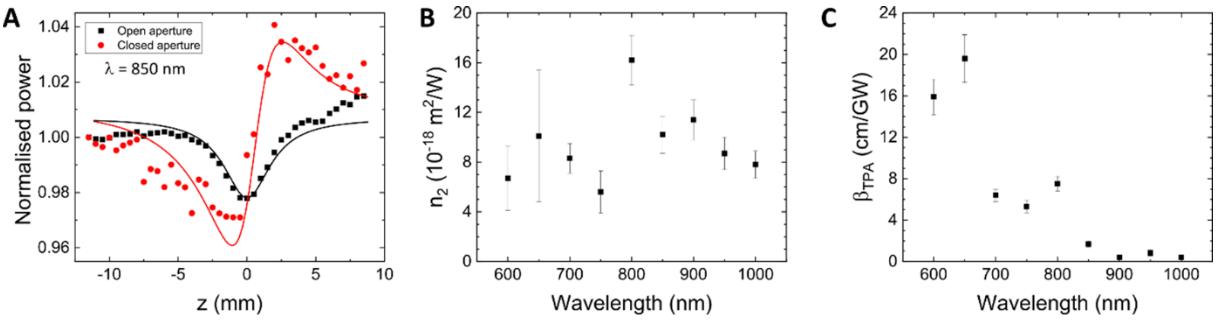

Fig. S2. Z-scan results of a double-side polished 350-µm thick GaP sample. (A) Representative open and closed aperture results at 850 nm wavelength. Solid lines are fits using Eq. S1 and Eq. S2. Nonlinear index **(B)** and two-photon absorption coefficient **(C)** against wavelength.

Numerical Simulations

The simulations were performed by means of the finite-difference time domain (FDTD) technique with the commercial software *Lumerical FDTD Solutions* and using the ellipsometric refractive index data for GaP. Pulses were linearly polarized Gaussian focuses at normal incidence inputted 3 µm above the air/GaP interface, considering a numerical aperture of 0.5 and with the focal position 3 µm below the air/GaP interface, in accordance with the experimental conditions. Perfectly-matched layers (PML) were used as the simulation boundary conditions to avoid reflections. To ensure all inter-pulse interactions were taken into account, the simulation length for all simulations was set to where the peak electric field in the GaP had dropped to one half, which corresponds to an intensity reduction to one quarter. A schematic representation of the simulation volume can be seen in Fig. S3.

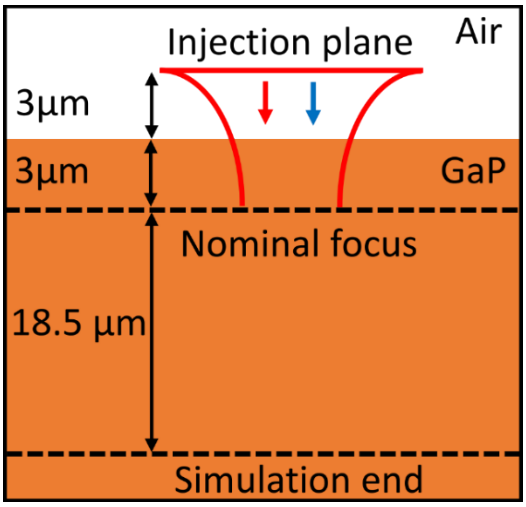

Fig. S3. Schematic representation of the numerical simulation volume depicting the ultrashort pulse injection plane, the nominal focus position, and the simulation length.

To estimate the strength of the nonlinear effect expected, we simulated first the differential reflectivity and differential transmissivity from the GaP sample as a function of the induced complex refractive index change at 800 nm (see Fig. S4). In this case, the simulations are purely linear and only compare how the reflectivity and transmission would change if a uniform refractive index change was induced evenly throughout the GaP sample. The numerical calculations show that differential reflectivity (Fig. S4A) would be dominated by the optical Kerr effect and differential transmissivity by two-photon absorption (Fig. S4B).

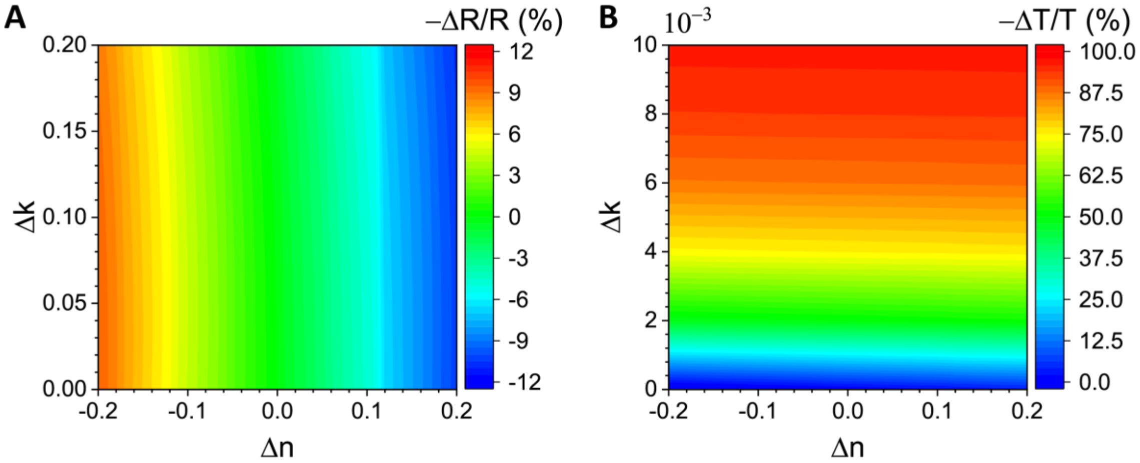

Fig. S4. Linear numerical simulations. Differential **(A)** reflectivity and **(B)** transmissivity at 800 nm wavelength with respect to changes in the complex refractive index of GaP.

To accurately model the ultrafast pulses used in the experiment, the experimental pulse spectra were used to create custom sources for the nonlinear simulations. The resultant pulse spectra used for the simulations (overlaid with the experimental spectrum) can be seen in Figure S5 along with the resultant time signals. These time signals are comparable to those experimentally determined as shown in previous work (*22*). The use of these custom sources caused ripples in the simulated differential transmissivity curves, which were removed by smoothing through adjacent averaging. Replacing the custom sources with standard Gaussian spectra sources of the same temporal duration smoothed the differential transmissivity curves but did not give good agreement with the experiment. In all cases the pump and probe pulses were inputted such that their peak electric field position reached the focus at the same time.

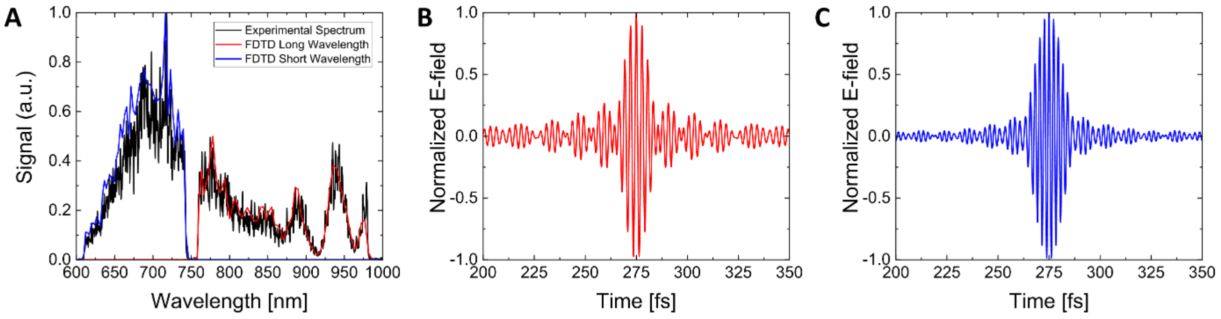

Fig. S5. Simulation of pump and probe pulses. **(A)** The experimental spectra of the long and short wavelength pulses overlaid by the spectra imported into the FDTD simulations, alongside the corresponding resultant time signals for the **(B)** long and **(C)** short wavelength pulses.

Finally, to model the optical Kerr effect and two-photon absorption in the GaP sample, the formulism presented by Suzuki (*26*) was followed to implement third order nonlinearities in the Yee FDTD algorithm, wherein the electric field E is calculated from the displacement field D and polarization P by $D = \varepsilon_0 E + P$. To simplify calculations, an instantaneous Kerr effect was assumed. In this approach, the polarization is divided into linear and nonlinear components as $P = P_{linear} + P_{Kerr} + P_{TPA}$, where the linear polarization is depended on the ellipsometric refractive index data for GaP. With an instantaneous Kerr effect, the nonlinear polarization due to the Kerr effect is $P_{Kerr} = \varepsilon_0 \chi_{Kerr} E$, where the nonlinear Kerr susceptibility is

$$\chi_{Kerr} = c_0 \varepsilon_0 n_0^2 n_2 |E|^2 \qquad \text{Eq. S3}$$

where c_0 is the speed of light in free space, ε_0 is the permittivity of free space, and $n_0 = 3.2$ is the refractive index of GaP at 800 nm.

For two-photon absorption, the nonlinear polarization is $P_{TPA} = \varepsilon_0 \chi_{TPA} E$, which is calculated using

$$\chi_{TPA} = -\frac{c_0^2 \varepsilon_0 n_0^2 \beta_{TPA}}{2i\omega} |E|^2 \qquad \text{Eq. S4}$$

The above nonlinear susceptibility must be discretized in the FDTD algorithm via the substitution of $-i\omega$ with $\partial/\partial t$

$$\chi_{TPA}^{n+1} = \chi_{TPA}^{n} - \frac{c_0^2 \varepsilon_0 n_0^2 \beta_{TPA} \Delta t}{2} \left|E^{n+\frac{1}{2}}\right|^2 \cong \chi_{TPA}^{n} - \frac{c_0^2 \varepsilon_0 n_0^2 \beta_{TPA} \Delta t}{4} [|E^{n+1}|^2 + |E^n|^2] \qquad \text{Eq. S5}$$

where n is the discretized step index and Δt is the time step. As χ_{TPA}^{n+1} depends on E^{n+1}, iteration is required for self-consistency.

Now, the equation to solve for the electric field can be rewritten as

$$\breve{D} = D - P_{linear} = \varepsilon_0 E \left[c_0 \varepsilon_0 n_0^2 n_2 |E|^2 - \frac{c_0^2 \varepsilon_0 n_0^2 \beta_{TPA}}{2i\omega} |E|^2 \right] \qquad \text{Eq. S6}$$

which again must be discretized via the substitution of $-i\omega$ with $\partial/\partial t$ and rearranged to solve for E^{n+1}

$$E^{n+1} = \frac{g_2(E^{n+1})}{g_1(E^{n+1})} E^n + \frac{\breve{D}^{n+1} - \breve{D}^n}{\varepsilon_0 g_1(E^{n+1})} \qquad \text{Eq. S7}$$

Where

$$g_1(E^{n+1}) = c_0 \varepsilon_0 n_0^2 n_2 |E^{n+1}|^2 + \frac{c_0^2 \varepsilon_0 n_0^2 \beta_{TPA} \Delta t}{8} [|E^{n+1}|^2 + |E^n|^2],$$

$$g_2(E^{n+1}) = c_0 \varepsilon_0 n_0^2 n_2 |E^n|^2 - \frac{c_0^2 \varepsilon_0 n_0^2 \beta_{TPA} \Delta t}{8} [|E^{n+1}|^2 + |E^n|^2] \qquad \text{Eq. S8}$$

As with Eq. S5, Eq. S7 must be iterated to achieve self-consistency. The maximum iteration in the nonlinear simulations was set to six, following that done elsewhere (*26*, *27*) to ensure accuracy without compromising simulation time.

By scanning the Kerr index and two-photon absorption coefficients for GaP across the wavelength spectrum as determined by the Z-scan method, approximate matches for the experimental differential transmissivity curves were found as seen in Fig. 3 of the main text.
